# Externally added cystatin C reduces growth of A375 melanoma cells by increasing cell cycle time

**DOI:** 10.1002/2211-5463.13162

**Published:** 2021-05-02

**Authors:** Hanna Wallin, Samar Hunaiti, Magnus Abrahamson

**Affiliations:** ^1^ Division of Clinical Chemistry & Pharmacology Department of Laboratory Medicine Lund University Sweden

**Keywords:** A375 cells, cell cycle, cell growth, cysteine peptidase, digital holographic microscopy, protease inhibitor

## Abstract

Some secreted cysteine protease inhibitors of the cystatin family appear to affect intracellular proteolysis and growth of human cells, as a result of internalization. Here, we studied the effects of external addition of the most abundant human cystatin, cystatin C, on viability and proliferation of cancer cells in culture. A dose‐dependent decrease in viable cells was seen for A375 melanoma, MCF‐7 breast cancer, and PC‐3 prostate cancer cells cultured in 1–5 µm cystatin C after 24 h. Real‐time assessment of growth rates in A375 cell cultures for 48 h by digital holographic microscopy showed an increased doubling time for cells cultured in the presence of 5 µm cystatin C (20.1 h) compared with control cells (14.7 h). A prolonged doubling time was already observed during the first 12 h, indicating a rapid general decrease in cell proliferation at the population level. Tracking of individual cells in phase holographic images showed that dividing cells incubated with 5 µm cystatin C underwent fewer mitoses during 48 h than control cells. In addition, the time between cell divisions was longer, especially for the first cell cycle. Incubation with the variant W106F‐cystatin C (with high cellular uptake rate) resulted in a lower number of viable cells and a prolonged doubling time than when cells were incubated with wild‐type cystatin C, but no effect was observed for (R24A,R25A)‐cystatin C (low cellular uptake). Thus, cystatin C causes prolonged cell division leading to decreased proliferation of melanoma cells, and internalization seems to be a prerequisite for this effect.

## Graphical abstract

Addition of cystatin C, a secreted cysteine protease inhibitor, to A375 melanoma cell cultures resulted in rapid decrease in cell numbers. Tracking of individual cells in phase holographic microscopy images demonstrated that cystatin C causes a prolonged cell cycle rather than increased cell death. Cystatin C variants with altered uptake properties indicated that internalization is needed for the proliferation effect.

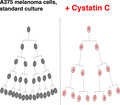

**Abbreviations**
DMSO: dimethylsulfoxide
ELISA: enzyme‐linked immunosorbent assay
MTT: 3‐(4,5‐dimethylthiazol‐2‐yl)‐2,5‐diphenyl‐tetrazolium bromide

There is a need to understand the complex interplay of proteases and their inhibitors in cancer. The activity of proteases, including cysteine cathepsins and legumain, is often misregulated in and around tumors [1]. The main family of natural inhibitors of the cysteine cathepsins B, C, F, H, K, L, O, S, V, W, and X (or Z), and legumain, are called cystatins and divided into three types. Type 1 cystatins (stefins) are cytosolic in location, type 2 cystatins are produced with a signal peptide and exported to the outside of the cell and type 3 cystatins are intravascular inhibitors with additional function to act as kinin precursors, the kininogens (reviewed in [2]). The most abundant of the human cystatins is cystatin C. It is a type 2 cystatin found in all body fluids and in almost all tissues and cells [3, 4].

All cystatins are inhibitors of papain‐like cysteine cathepsins (Family C1 [5]), and cystatin C has the most general inhibition profile with *K*_i_ values in the nanomolar range for virtually all cysteine cathepsins [3]. In contrast to most of the other cystatin family members, cystatin C has a second enzyme binding site as well resulting in high affinity for another type of enzyme, legumain (Family C13 [5, 6]). These properties mean that cystatin C has capacity to control the activities of a dozen potent proteases, which normally are located in lysosomes or endosomal vesicles and have optimal proteolytic activities at acidic pH. If these enzymes are leaking out from dying cells, the high concentration of cystatin C in extracellular fluids will lead to efficient down‐regulation of their activities, normally within milliseconds [3].

There is ample evidence from gene silencing studies in mice and clinical data that cystatin C and other cystatins have multiple effects *in vivo* [7]. Concerning direct evidence in cell systems, external addition of cystatin C has been shown to block replication of human herpes simplex and corona virus in cells [8, 9]. In addition, human cystatin C and its analogue in chicken have shown protective effects against cancer cell invasion and parasite infections [10, 11, 12, 13]. Furthermore, an increasing body of evidence demonstrates that cystatins can regulate cell proliferation [14, 15, 16, 17], protect against oxidative stress that induces cell death [18] and can affect cytokine release [19, 20, 21]. Therefore, cystatin C must be regarded as a molecule with pleiotropic effects having impacts on an array of both normal and patho‐physiological processes *in vivo*.

Some cystatin C effects on cell behavior could be the result of receptor‐mediated interference with signaling pathways [22, 23] and others due to internalization of the inhibitor followed by inhibition of intracellular target enzymes (cysteine cathepsins or legumain) involved in the respective processes [24, 25, 26]. In a recent study, we observed that human cystatins C and D enhanced apoptosis induced by hydrogen peroxide (H_2_O_2_) and decreased proliferation of leukemic cells [17]. However, other studies have concluded that there is little or no effect on proliferation of human cells by cystatin C [13]. The present investigation was undertaken to closer examine whether externally added cystatin C has ability to affect cancer cell proliferation and, hence, potential to suppress tumor growth.

## Results

### Reduced number of viable cells after incubation with external cystatin C

**Fig. 1:**
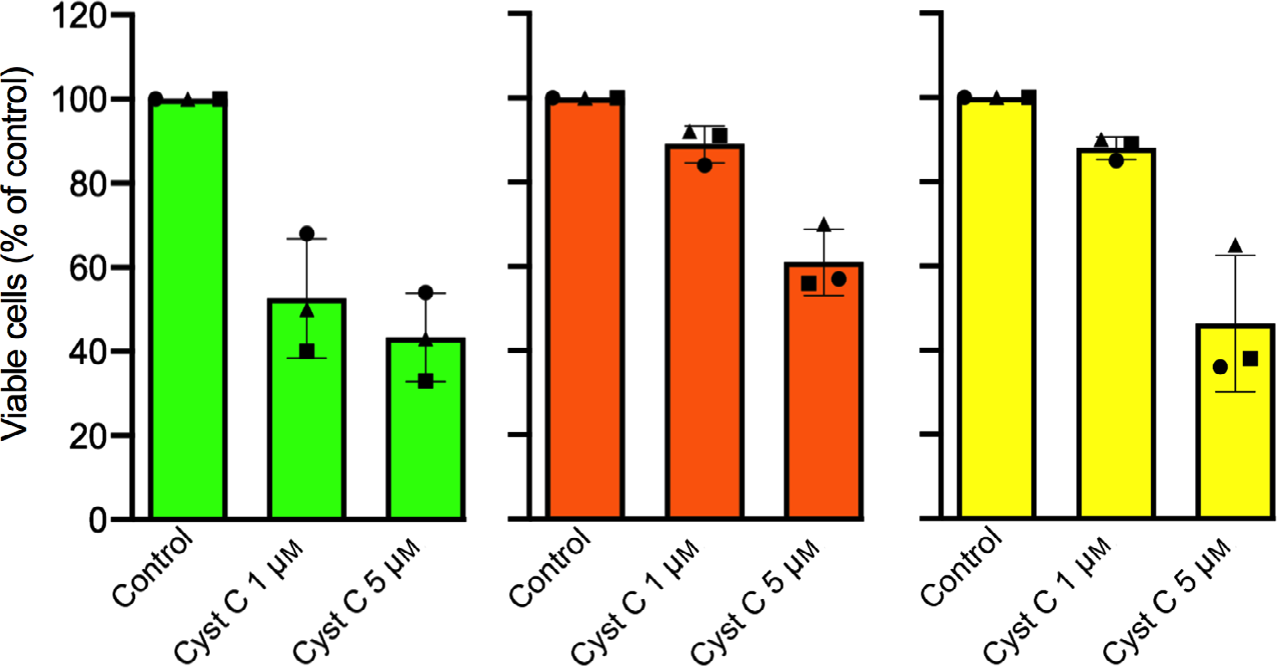
Effects of externally added cystatin C on viability of cultured melanoma and epithelial cancer cells. A375 melanoma (*green bars*), MCF‐7 breast cancer (*orange*) and PC‐3 prostate cancer (*yellow*) cells were incubated with medium containing 0 (*Control*), 1 or 5 µm cystatin C for 24 h. The experiments were performed three times with 4–6 wells each time. Statistics were calculated on raw data in each experiment. For all three experiments and A375 cells, both 1 and 5 µm cystatin C, *P* < 0.005; MCF‐7, 5 µm *P* < 0.05; PC‐3, 5 µm *P* < 0.05. Bars represent the mean of the mean values from the three experiments, with error bars denoting SD. *Circles*, *squares*, and *triangles* represent the mean results from each experiment. The result from the control cells in each experiment was set to 100%, and the rest of the values were correlated to that.

We recently observed a dose‐dependent decrease in viability when cystatin C was added to the medium of U937, Jurkat, and HL‐60 leukemia cell cultures [17]. To study this further in other cell types, we initially addressed A375 melanoma cells. 3‐(4,5‐dimethylthiazol‐2‐yl)‐2,5‐diphenyl‐tetrazolium bromide (MTT) assay was performed to study the effect on viable cell count after incubation with recombinant human cystatin C. Following seeding and attachment, the cells were incubated in medium containing 0 (control), 1 or 5 µm cystatin C for 24 h. A decreased number of viable cells was observed for cells in 1 µm cystatin C (53% of control) and when 5 µm cystatin C was used the number decreased further (43%; Fig. 1, green bars). Identical experiments were made with breast cancer MCF‐7 cells (Fig. 1, orange bars) and prostate cancer PC‐3 cells (Fig. 1, yellow bars). A slightly smaller but similar dose‐dependent pattern of decreased viable cell counts was seen also for these epithelial cell lines (Fig. 1).

### Growth of A375 cells in culture after incubation with external cystatin C

**Fig. 2:**
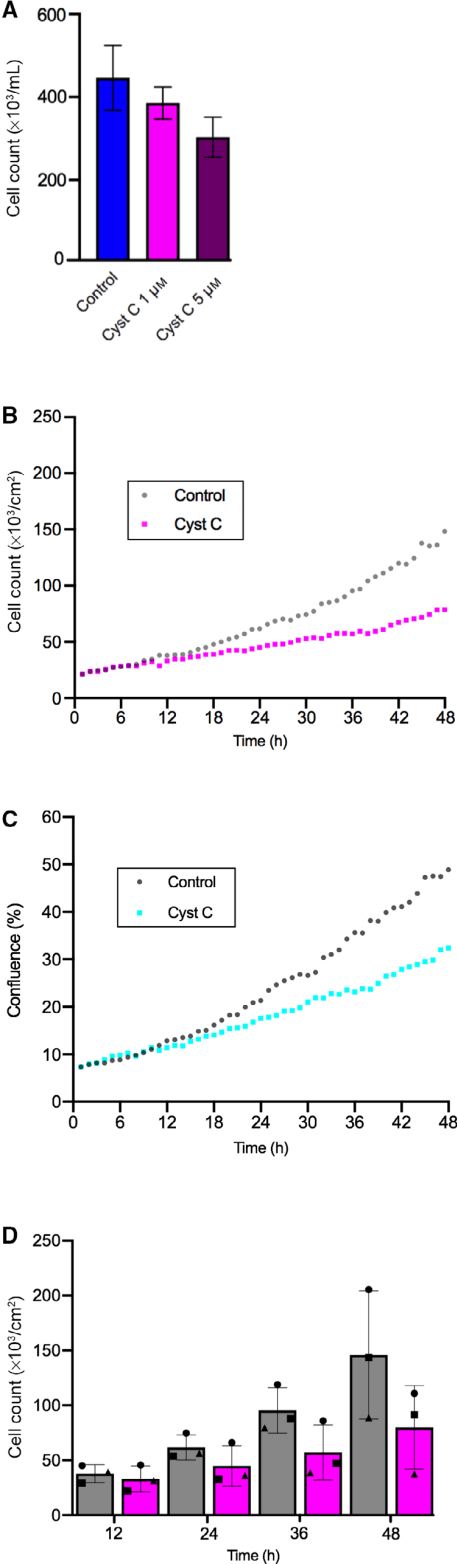
Effects of externally added cystatin C on growth of A375 melanoma cells. (A) A375 cells were incubated with medium containing 0 (*Control*), 1 or 5 µm cystatin C for 24 h, followed by counting in a hemocytometer. Results from two independent experiments with two wells of each treatment are shown, with error bars indicating SD. (B, C) A375 cells were incubated with medium containing 0 (*Control*) or 5 µm cystatin C for 48 h and phase holograms were captured every hour. The AppSuite software (Phase Holographic Imaging) was used to draw growth curves based on normalized cell counts (B) and normalized confluence (C). Each point shown represent the mean value from three independent experiments, with 2 positions analyzed in each of 8 wells in each experiment. (D) Selected timepoints from the growth curve in (B). Statistical analysis was performed on raw data to assess the significance of differences for cells cultured with 5 µm cystatin C compared with control within each experiment. Exp 1: 12 h, *P* < 0.02; 24 h, *P* < 0.07; 36 h, n.s.; 48 h, *P* < 0.05. Exp 2: 12 h, *P* < 0.001; 24 h, *P* < 0.001; 36 h, *P* < 0.005; 48 h, *P* < 0.001. Exp 3: 12 h, *P* < 0.06; 24 h, *P* < 0.005; 36 h, *P* < 0.04; 48 h, *P* < 0.004. Bars represent the mean of the mean values from the three experiments, with error bars denoting SD. *Circles*, *squares*, and *triangles* represent the mean results from each experiment.

The melanoma A375 cells were chosen for continued in‐depth studies of the cellular effects of cystatin C, as the reduction in viable cell numbers by externally added cystatin C was largest on these cells, but also because of their growth properties making them suitable for study by digital holographic microscopy. Initially, attached A375 cells were incubated in medium without or with addition of cystatin C for 24 h and then detached and counted in a hemocytometer (Fig. 2A). At this timepoint, the control cells had more than doubled in number, as the 200 000 cells seeded had increased to 446 000 ± 78 000 (mean value ± SD), while incubation with 1 µm cystatin C resulted in decreased cell number (385 000 ± 39 000) compared with the control cells. Incubation with 5 µm cystatin C showed an even more pronounced effect as the cell number reached only two‐thirds of the number of the control cells (303 000 ± 48 000).

For detailed studies of the growth of A375 cell populations, we used digital holographic imaging to follow the cultures in real‐time in the CO_2_ incubator [27]. Holographic images were taken every hour during 48 h, cells were defined and counted in each image to draw growth curves (Fig. 2B). Presence of 5 µm cystatin C reduced the endpoint cell number to 55% (81 000 ± 37 000; mean value ± SD) compared with control cells (146 000 ± 58 000). The cell density at the start of the experiment corresponded to 7% confluence, which increased to 49% for control cells but only to 33% for parallel cultures with externally added cystatin C, agreeing well with a reduced proliferation rate when cystatin C was present (Fig. 2C). However, under the conditions of our experiment, assessment of growth curves from cell counts gave higher reproducibility than confluence curves when automated counting was used. A trend toward reduced cell counts in cultures incubated with cystatin C was seen in three independent experiments already after 12 h and continued thereafter for all chosen 12 h intervals (Fig. 2D).

Data from the mean cell count results shown in Fig. 2B was used to manually calculate the population growth rate after addition of 5 µm cystatin C to the A375 cell cultures. Calculated over the entire 48 h culture time, the mean growth rate was 0.0344 per hour when cystatin C was added, compared with 0.0470 per hour for control cells (Table 1). This corresponds to an increased doubling time in the presence of cystatin C in the medium from 14.7 to 20.1 h. Sub‐analysis of 12 h time periods indicated a prolonged doubling time already during the first 12 h of culture when cystatin C was included in the medium (13.8 rather than 12.1 h). Doubling times assessed from later time intervals of the growth curve were considerably longer, but even more prolonged in cystatin C containing medium (Table 1).

**Table 1:** Growth rates and doubling times of A375 cells cultured without or with 5 µm wild‐type cystatin C or its variants W106F‐ and (R24A,R25A)‐cystatin C

	Growth rate × 10^3^ (h^–1^)				Doubling time (h)			
Interval (h)	Control	WT–	(R24A,R25A)–	W106F–	Control	WT–	(R24A,R25A)–	W106F–
0–48	47.0 ± 1.8	34.4 ± 13.2	54.3 ± 15.8	28.9 ± 13.2	14.7	20.1	12.8	24.0
0–12	57.1 ± 15.1	50.3 ± 8.5	73.0 ± 51.3	47.3 ± 6.2	12.1	13.8	9.5	14.7
12–24	46.6 ± 2.5	32.8 ± 12.2	52.0 ± 6.6	27.5 ± 13.5	14.9	21.1	13.3	25.3
24–36	43.4 ± 3.2	28.7 ± 9.4	47.4 ± 3.3	22.1 ± 16.2	16.0	24.2	14.6	31.4
36–48	41.0 ± 5.5	25.9 ± 10.0	44.9 ± 1.8	18.8 ± 16.6	16.9	26.8	15.4	37.0

Raw cell count data every hour for 48 h from 2 to 3 independent experiments were used to calculate mean growth rates and doubling times for 12‐h increments, as detailed in the Methods section. The variation between individual experiments is indicated by SD. The same data was also used to draw mean growth curves (Figs 2B and 8A).

**Fig. 3:**
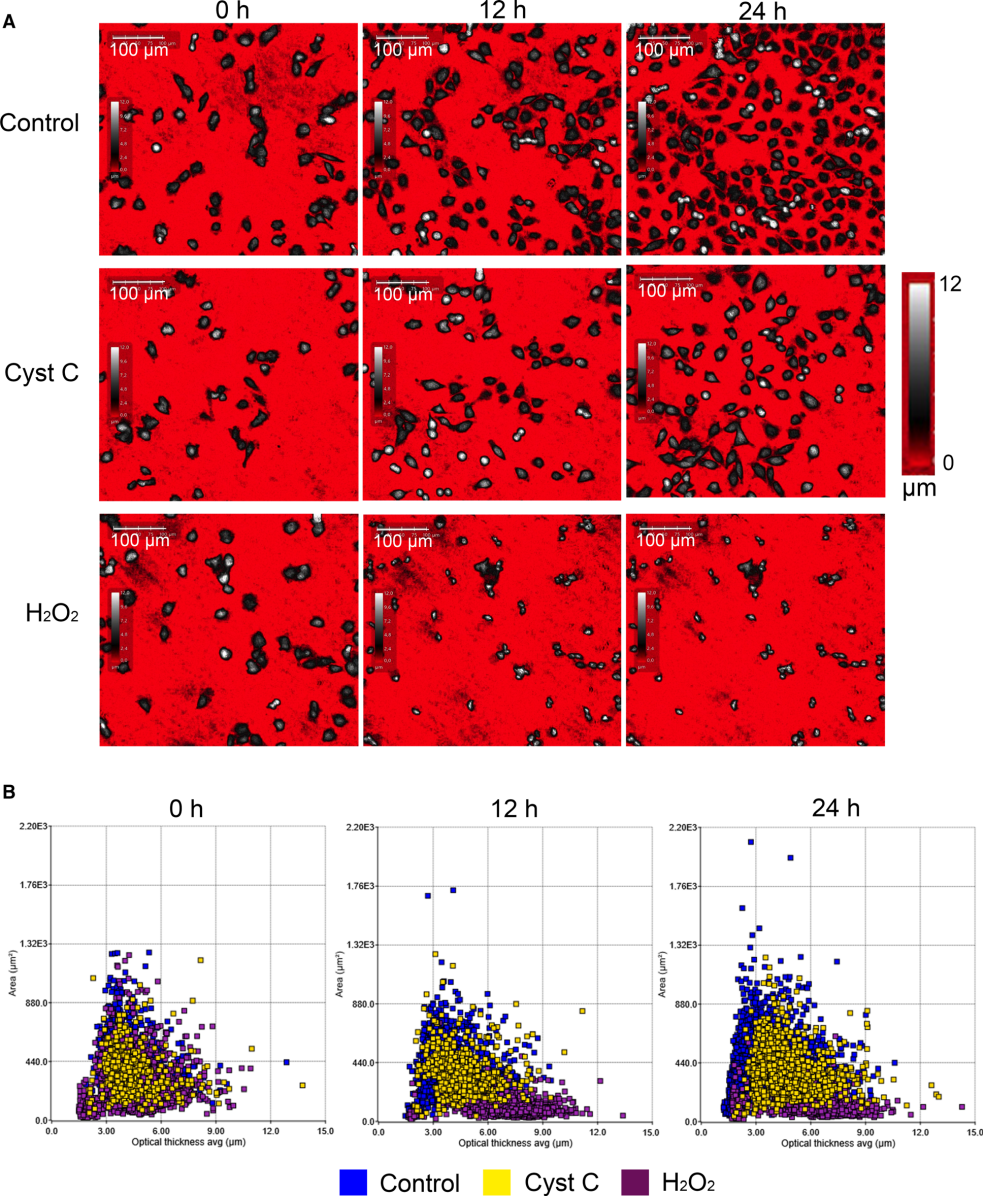
Morphology of cells treated with H_2_O_2_ to induce cell death or with cystatin C. (A) A375 cells were incubated with H_2_O_2_ or cystatin C and digital holographic microscopy images were captured after 0, 12, and 24 h. Cell thickness is shown by color‐coding, as shown with the color bar to the right. The images shown are representatives of 16 images analyzed per condition. *Scale* *bars*, 100 µm. (B) Scatter plots of the area (µm) versus the average optical cell thickness (µm) for all cells in the 16 holograms at each timepoint per each condition.

The reduced cell numbers and prolonged doubling times in the presence of cystatin C could be due to either a decreased proliferation rate or increased cell death. Analysis of cell death by measuring cell area and optical thickness by phase holographic microscopy has been conducted previously for TUNEL positive neuroblastoma cells [28]. To address the possibility that cystatin C is inducing cell death, we studied the morphology of cells cultured with or without cystatin C in phase hologram images. A reduced cell number in cultures with cystatin C was apparent compared with control after 12 and 24 h, but the cells looked healthy and continued to proliferate throughout the entire experiment (Fig. 3A). A375 cells cultured in the presence of 100 µm H_2_O_2_ (known to induce death of A375 cells [29]), in contrast, looked condensed with a smaller area and did not proliferate (Fig. 3A). Essentially all cells treated with H_2_O_2_ displayed a small area and high thickness after 12 h (Fig. 3B). On the other hand, the majority of cells in the population incubated with cystatin C were similar to control cells with respect to area and thickness. This suggested that cystatin C decreases the A375 proliferation rate without increasing cell death.

### Real‐time analysis of cell division in A375 cultures incubated with external cystatin C

**Fig. 4:**
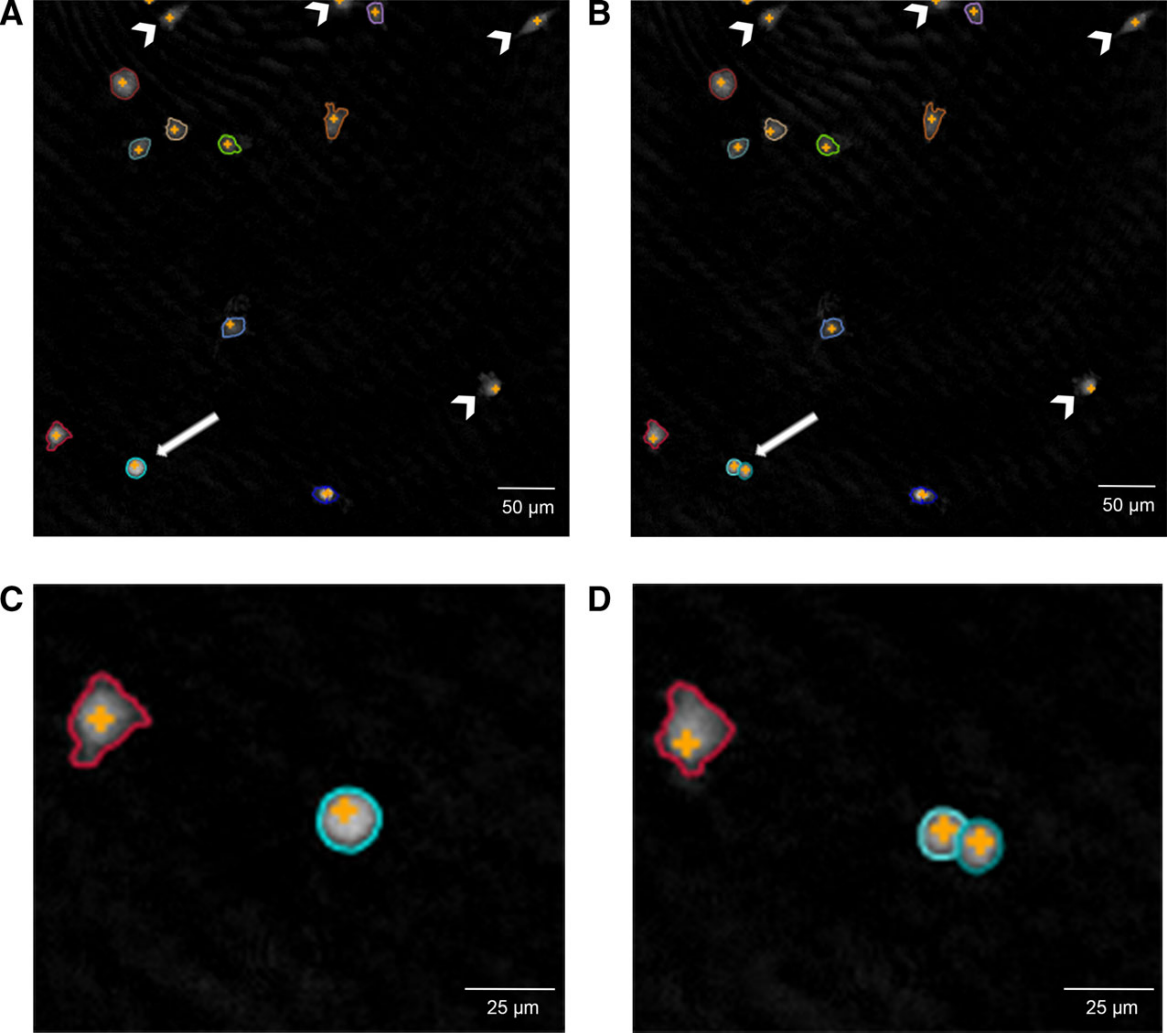
Digital holographic images of A375 cells in culture. A375 cells growing in culture were visualized every 5 min by phase holographic microscopy. Cells in images were defined by the HStudio software (Phase Holographic Imaging) and selected for tracking after manual inspection. (A, B) Images of the same position captured 5 min apart. The *arrowheads* indicate cells that were excluded from analysis, as they were migrating out of the image area before the first cell division. The *arrow* shows a cell that has rounded up for division in (A) and has divided 5 min later in (B), which is shown in higher magnification in (C) and (D). *Scale* *bars*, 50 µm in (A, B), 25 µm in (C, D).

To further study the effects on proliferation seen at the cell population level, we followed the fate of individual A375 cells in the presence or absence of 5 µm cystatin C in the culture medium. Real‐time holographic images were captured every 5 min for 48 h, in three randomly selected areas in each culture well (two wells with addition of cystatin C and two wells without). All cells in the first image of each experiment were identified by visual inspection (130 control cells and 136 from cultures with cystatin C) and tracked until next division (see Fig. 4 for example). Cells that migrated out of the image area before the first division were omitted. The resulting daughter cells of each cell division were labeled and tracking continued in later images, which made it possible to construct generation trees and to calculate the time between cell divisions for the individual cells.

**Fig. 5:**
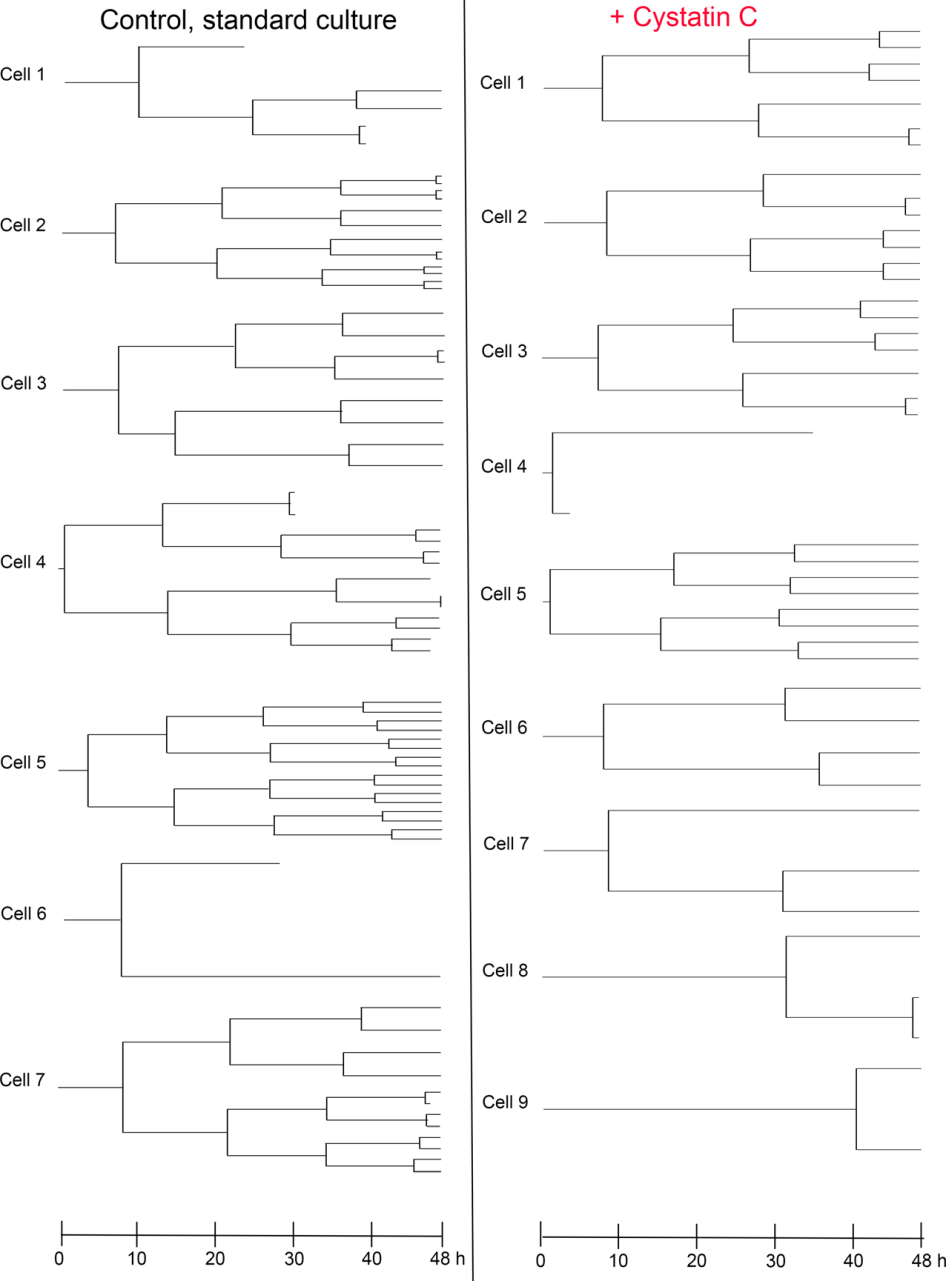
Generation trees for A375 cells grown in the presence or absence of cystatin C. A375 cells were incubated with medium containing 0 (*Control*) or 5 µm cystatin C for 48 h and phase holograms were captured every 5 min. All cells in the first image were identified by visual inspection and tracked until next division. The resulting daughter cells of each cell division were labeled and tracking continued in later images. Data from one position in a control well and one well with cystatin C containing medium were analyzed and presented as generation trees. In total, 130 control cells and 136 cystatin C‐treated cells were analyzed.

**Fig. 6:**
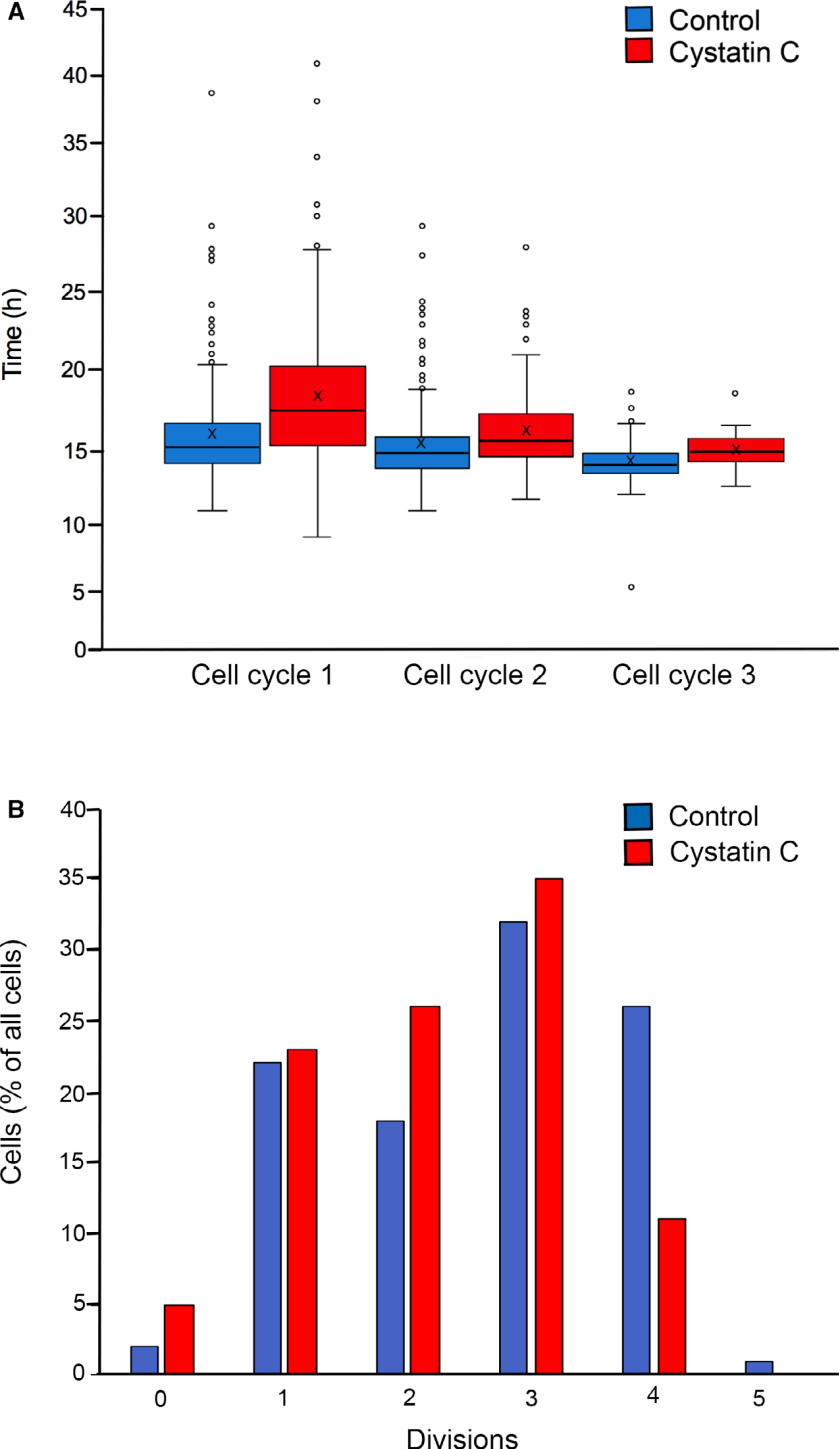
Externally added cystatin C increases cell cycle time and decreases the number of A375 cell divisions. (A) Data from cell tracking were used to determine the length of individual cell divisions, for A375 cells cultured in the absence (*Control*) or presence of 5 µm cystatin C. The box plot shows the central 50% of the populations, whiskers 25% each, solid line median value and X denotes the mean value. Statistical analysis to compare cells without and with cystatin C for cell cycle 1 (*n* = 186 and 173, respectively), *P* < 0.001; cell cycle 2 (*n* = 219 and 174), *P* < 0.001; cell cycle 3 (*n* = 144 and 34), *P* < 0.001. (B) The relative numbers of cell divisions observed during 48 h for all tracked cells in the first timepoint of the experiment are presented.

Examples of generation trees are shown in Fig. 5, for A375 cells grown in the absence (left) or presence of cystatin C in the medium (right). The cell cycles were longer when the A375 cells were cultured in the presence of cystatin C and the cell divisions were fewer. Applicable for all tracked cells, we observed that the numbers of cell divisions during the time of the experiment differed significantly between individual cells and it was also apparent that the cell cycle time varied considerably (Fig. 6). It was noticeable that some cell divisions were more than twice as long as the median cell cycle length, especially in cell cycle 1 and when cells had been grown in the presence of cystatin C. The mean time between cell divisions was 15.3 ± 3.7, 14.3 ± 2.8, and 13.2 ± 1.4 h for the first, second, and third cell cycle of the control cells, respectively (Fig. 6A). For cells cultured in the presence of 5 µm cystatin C, the mean time between divisions was 18.0 ± 4.6, 15.8 ± 2.8, and 14.2 ± 1.2 h. At comparison, the difference was significant for all three cell cycles with *P* values <0.001.

There was also a substantial variation in the number of completed cell cycles in the 48 h cultures when all tracked cells were analyzed. Some cells migrated fast and moved out of the image area or aggregated with others and could thus not be followed during the entire time period. A few cells (<5%) did not move or divide at all and others had short cell cycles and divided more often. The most common was 3 completed cell divisions, regardless of cystatin C addition to the medium or not (Fig. 6B). However, in the presence of cystatin C, just 11% of the tracked cells managed to divide 4 times, whereas 4 divisions were seen for 26% of the control cells and even 5 divisions for a few cells (Fig. 6B).

### Cellular uptake of cystatin C is needed for effects on A375 cell proliferation

**Fig. 7:**
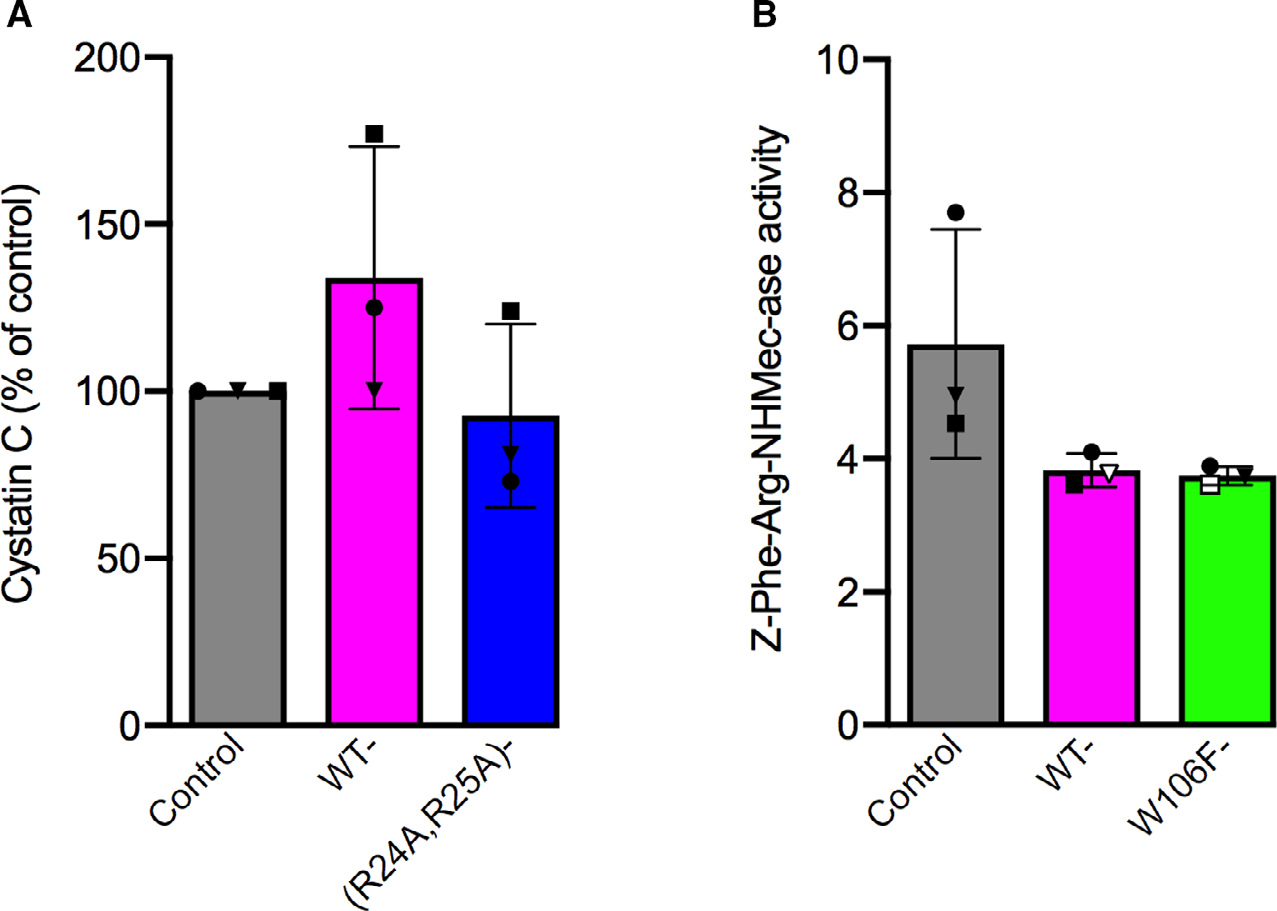
Cellular uptake of cystatin C variants and their inhibition of intracellular cysteine cathepsin activity. (A) A375 cells were incubated for 6 h with 1 µm wild‐type or (R24A,R25A)‐cystatin C. Intracellular cystatin C content was measured by ELISA. The results were correlated to the protein content of each lysate, and compared with control. *Bars* represent mean values from three independent experiments, with *error bars* denoting SD, and the *symbols* show the mean results from each experiment. (B) Cells were incubated with medium containing 1 µm wild‐type or W106F‐cystatin C for 6 h. Intracellular cysteine cathepsin activity was measured using the general substrate for papain‐like endoproteases, Z‐Phe‐Arg‐NHMec. Activity was expressed as FU/min/mg cellular protein as detailed in the Methods section. *Bars* indicate mean values from three independent experiments, with *error bars* denoting SD, and the *symbols* show the mean results from each experiment.

Cystatin C is known to get internalized by various cells including A375 cells [30]. To elucidate whether the internalization is a prerequisite for the observed effects on cell proliferation, we analyzed two cystatin C variants, (R24A,R25A)‐ and W106F‐cystatin C with different uptake properties. Both are as active as legumain inhibitors as wild‐type cystatin C is, but W106F‐cystatin C shows moderate (10–100‐fold) decreased affinity for cysteine cathepsins [26, 31]. For the A375 cells, uptake of W106F‐cystatin C corresponded to more than doubled levels of intracellular cystatin C after 6 h [30]. As no corresponding data were available for (R24A,R25A)‐cystatin C, we analyzed the uptake of this variant in similar experiments (Fig. 7A). As in MCF‐7 cells [26], there were no signs of significant uptake into A375 cells after 6 h of incubation. The uptake of wild‐type or W106F‐cystatin C was paralleled by inhibition of intracellular cysteine cathepsin activity measured by the general endopeptidase substrate, Z‐Phe‐Arg‐NHMec (Fig. 7B). Inhibition of intracellular legumain activity in these cells after uptake of cystatin C has previously been shown [30].

**Fig. 8:**
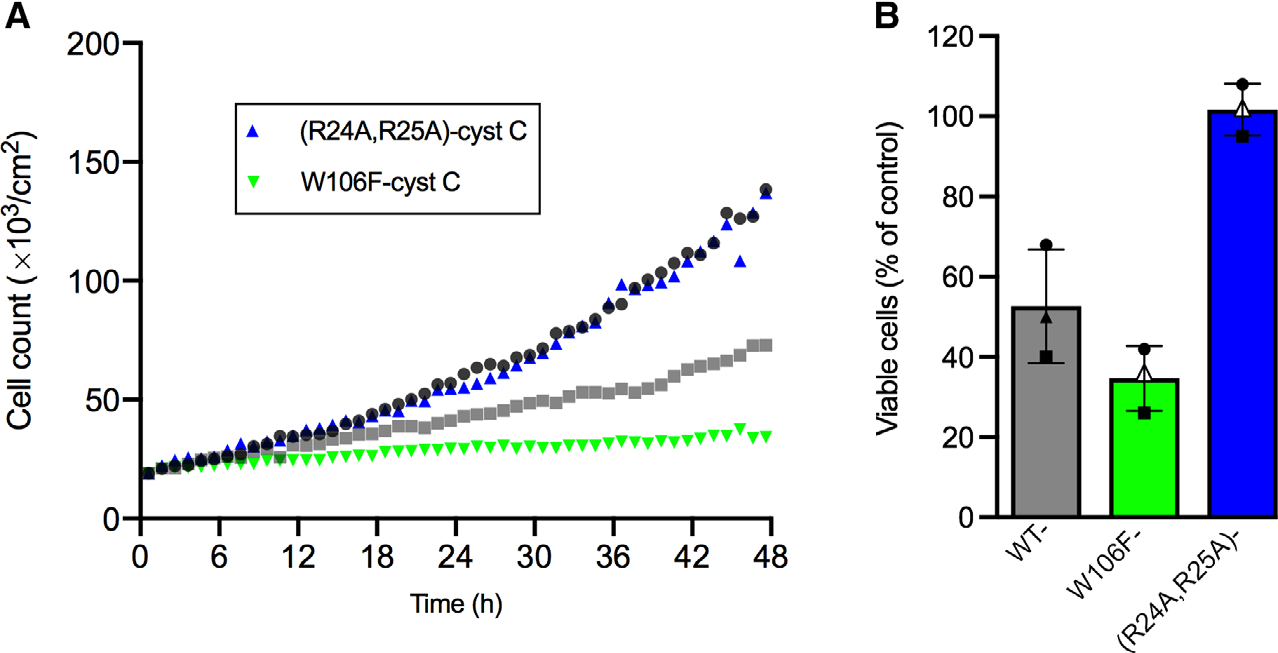
Effects of externally added cystatin C variants on growth and viable cell count. A375 cells were incubated with medium containing 5 µm W106F‐ or (R24A,R25A)‐cystatin C for 48 h. (A) Phase holograms were captured every hour in 2 positions of 8 wells for each condition, in duplicate experiments. Mean growth curves based on normalized cell counts were drawn and compared with curves for 0 (*black* *circles*) and 5 µm wild‐type cystatin C (*gray squares*) (Fig. 2B). (B) End‐point viable cell counts were measured by MTT assay for the cystatin C variants and compared with results for 0 (control) and 5 µm wild‐type cystatin C (Fig. 1). The experiment was performed three times with 6 wells each time. Statistics were calculated on raw data in each experiment; wild‐type compared with W106F‐cystatin C, n.s.; wild‐type compared with (R24A,R25A)‐cystatin C, *P* < 0.003. Bars represent the mean of the mean values from the three experiments with the two cystatin C variants, with error bars denoting SD. *Circles*, *squares*, and *triangles* represent the mean results from each experiment.

A375 cells were incubated in the presence or absence of 5 µm of either wild‐type cystatin C or the variants (R24A,R25A)‐ or W106F‐cystatin C for 48 h. Real‐time holographic images were captured every hour as before and growth curves based on cell counts were drawn (Fig. 8A). The results show that the number of cells at the end of the experiment was equal for the cells incubated with (R24A,R25A)‐cystatin C (145 000 ± 42 000; mean value ± SD) and the control cells (146 000 ± 58 000). The cells incubated with W106F‐cystatin C displayed an almost flat growth curve and the number of cells after 48 h only reached 35 000 ± 810 (24% of control). During the entire 48 h period the mean population growth rate for cells incubated with (R24A,R25A)‐cystatin C was 0.0543 per hour and 0.0470 per hour for the control cells (Table 1). The W106F‐cystatin C incubated cells had a growth rate of 0.0289 per hour, compared with 0.0344 per hour for cells incubated in medium containing the wild‐type inhibitor. These growth rates correspond to doubling times of 14.7 h for the control cells and 20.1, 12.8, and 24.0 h for cells incubated in wild‐type‐, (R24A,R25A)‐, and W106F‐cystatin C, respectively (Table 1). The sub‐analysis of 12 h intervals indicated a more prolonged doubling time caused by W106F‐ than wild‐type cystatin C already the first 12 h (14.7 and 13.8 h, respectively), which was more pronounced at later 12‐h increments. On the other hand, the cells incubated with (R24A,R25A)‐cystatin C showed almost the same doubling times as nontreated cells in all 12 h intervals (Table 1).

Both growth rate and the number of viable A375 cells were decreased after addition of 5 µm W106F‐cystatin C to the cell cultures compared with cells incubated with wild‐type cystatin C. In comparison, when cells were incubated in the presence of the variant with a low rate of internalization, (R24A,R25A)‐cystatin C, both the growth rate (Fig. 8A) and the number of viable cells (Fig. 8B) were similar to the control.

## Discussion

Cellular uptake of cystatin C [25, 32] and other secreted cystatins like cystatin D [17, 33], cystatin E/M [30, 34] and cystatin F [35, 36] in sufficient amounts to affect intracellular processes seems to be well established. Our research approach has been to explore what the therapeutic potential of recombinant cystatin C could be, by externally adding it to cell cultures in concentrations that are physiologically reasonable [3].

In the present paper, we have studied three different tumor‐derived epithelial cell lines and observed that externally added cystatin C caused a reduction in cell numbers, similar to its effect on leukemia cells [17]. Moreover, A375 melanoma cells were studied both individually and at the population level by digital holographic microscopy. This allowed us to conclude that cystatin C causes a decreased proliferation by slowing down the cell cycle and that internalized cystatin C is responsible. This effect on cell division was already noticeable after 12 h which indicates a rapid decrease in the growth rate rather than 'toxic' effects resulting in increased cell death. In fact, inspection of holographic images during the entire experiment did not reveal any signs of apoptotic or dead cells in the cultures incubated with cystatin C (Fig. 3). According to previous confocal microscopy results for MCF‐7 and A375 cells, internalized cystatin C is found in endo‐lysosomal‐like vesicles within hours [26, 30], agreeing well with this rapid effect on cell division or growth. In the present study, we also demonstrated an even more suppressed cell proliferation by a cystatin C variant showing higher uptake rate than wild‐type cystatin C, and no suppression by an uptake‐negative variant, which suggests that intracellular inhibition of a target enzyme is at hand. At present, we do not know which of the 12 endo‐lysosomal cysteine proteases could be the target for inhibition by internalized cystatin C. Concerning a possible involvement of lysosomal cysteine cathepsins in proliferation, it has been reported that the cell‐permeable cathepsin B inhibitor CA‐074Me can reduce proliferation of MCF‐10 cells and also reduce the tumor size in 3D culture [37]. Interestingly, a recent report shows that cathepsin B can leak from lysosomes and contribute to chromosome segregation at cell division in MCF‐7 cells [38]. Internalization of cystatin C could mean that lysosomal cysteine proteases like cathepsin B are down‐regulated and inactivated [25, 26] already before leakage. However, another recent publication provides evidence that not only cathepsin B but also other lysosomal cysteine proteases such as cathepsin C, L, and S can escape from lysosomes in U937, THP‐1, and U‐87‐MG cells [7]. Cystatin C is a potent inhibitor of all the cysteine cathepsins *in vitro* [3] and our present data (Fig. 7B) show that the magnitude of cystatin C uptake is sufficient to inhibit intracellular cysteine cathepsin activity. The substrate used does not discriminate between different cysteine cathepsins with endoprotease activity, so it remains to investigate which target enzyme is involved in the mechanism leading to slowed‐down division of A375 cells.

The variant W106F‐cystatin C had a drastic effect on A375 cell growth and viability, while the noninternalized variant (R24A,R25A)‐cystatin C had no effect at all (Fig. 8). On the contrary, the results from a previous study showed that externally added (R24A,R25A)‐cystatin C reduced viable cell counts of three different leukemic cell lines although not as pronounced as for wild‐type or W106F‐cystatin C [17]. A possible explanation to this divergency may be different cystatin uptake pathways and rates depending on cell type, and it is known that leukemic cells has a quite specific expression pattern of cysteine proteases and their inhibitors [39, 40, 41].

We used digital holographic microscopy for in‐depth real‐time studies of cell proliferation and death. The resolution is lower than in fluorescence microscopy methods, yet it allows continuous studies of cell behavior in culture for several days without any staining or harmful laser light [42]. The A375 cells were chosen because of their appropriate morphological and growth properties (growth in monolayers with relatively short generation time, smooth surface with defined borders and without protrusions). An advantage with the method is the possibility to study live images of individual cells in long‐time culture. This enabled us to see a remarkable variation in both cell cycle time and morphology within the A375 cell population, that we previously assumed was homogeneous. Using the cell tracking function of the software we could quantify the number of cell divisions of individual cells (Fig. 6), and construct generation trees (Fig. 5). This approach has earlier been used to successfully study the effects of cytotoxic drugs in various cell lines [43]. We could also see that the cells displayed a variation in the time to complete the cell cycle, as the time in M phase from rounding up (prometaphase) to cytokinesis could be long compared with the normal behavior. It would have been interesting to study the internalization of cystatin C in parallel, to determine whether there is a correlation between uptake and marked delay in M phase in individual cells.

In conclusion, the present study of individual cell divisions supported by population‐based data strongly indicates that cystatin C affects A375 cell growth by prolonging the cell cycle time. Thus, cystatin C has ability to regulate cancer cell proliferation and, hence, potential to suppress tumor growth.

## Materials and methods

### Cell culture

A375 melanoma cells, PC‐3 prostate cancer cells, and the human breast adenocarcinoma cell line MCF‐7 were all purchased from American Type Culture Collection (ATCC, Manassas, VA, USA). The cells were cultured in Dulbecco's Modified Eagle's Medium with 4500 mg/L glucose, GlutaMAX–I, and pyruvate supplemented with 100 IU·mL^−1^ penicillin/streptomycin and 10% fetal bovine serum. Cell media, cell culture consumables, antibiotics, trypsin‐EDTA, fetal bovine serum and PBS were purchased from Thermo Fisher Scientific Inc. (Rockford, IL, USA) unless otherwise stated.

### Production, purification, and characterization of proteins used for cell culture studies

Recombinant wild‐type cystatin C was expressed in *Escherichia coli*, purified, and characterized according to protocols described in detail elsewhere [44, 45]. The W106F‐ and (R24A,R25A)‐cystatin C variants were produced by site‐directed mutagenesis and expressed, purified, and characterized in a similar way as wild‐type cystatin C, as detailed earlier [26, 31]. Finally, the recombinant proteins were purified by Detoxi‐Gel™ Endotoxin Removing Gel (Pierce, Rockford, IL, USA). Protein concentrations were determined by Coomassie Protein Assay (Thermo Fisher Scientific) and by measurement of A_280_ (NanoDrop 2000, Thermo Fisher Scientific). All recombinant proteins used were at least 90% pure and fully active as protease inhibitors according to papain titration [46].

### Cell count in hemocytometer

A375 cells (200 000) were seeded in wells of a 6‐well plate and allowed to attach for 24 h. Then, the medium was exchanged for fresh medium containing 0, 1 or 5 µm wild‐type cystatin C and incubated for another 24 h before the cells were detached using 0.05% trypsin‐EDTA. Cells were counted in a Bürker chamber according to general protocols.

### MTT viability assay

A375 cells were seeded in 96‐well cell culture plates at a density of 2,000 cells/well, allowed to attach for 24 h, before incubation with medium containing 0, 1 or 5 µm wild‐type cystatin C or the variants (R24A,R25A)‐ or W106F‐cystatin C for 48 h. The experiment was performed three times with 4–6 wells for each condition in every experiment. After the 48 h incubation 5 mg/mL MTT dye (Sigma‐Aldrich Chemie, Steinheim, Germany) was added to the wells, followed by a 4 h incubation step in 37 °C. Living cells convert the MTT reagent to blue water‐insoluble crystals of formazan which were dissolved with 100% DMSO (Sigma‐Aldrich). Finally, the absorbance was measured in a microplate reader at 540 nm [47, 48]. The absorbance of the control cells was set to 100% in each experiment and the other results were correlated to that.

### Digital holographic microscopy

Digital phase holographic microscopy was performed using a HoloMonitor M4 (Phase Holographic Imaging AB, Lund, Sweden), allowing real‐time monitoring of 3D images of cells in culture. In the quantitative phase imaging HoloMonitor system, a low‐power laser is split into two beams with an object beam illuminating the cell sample to create a phase shift in the laser beam and a reference beam that is kept undisturbed [42]. When the two beams are merged they create an interference pattern, the hologram, which is projected on the camera and recorded by a digital image sensor. These images can then be used for a computer‐processed analysis of different cellular processes such as cell proliferation, motility, and division without requirement for any cell staining or labeling [43]. The HoloMonitor M4 was installed inside a standard cell culture incubator with water‐saturated atmosphere at 37 °C in 5% CO_2_ prior to the experiments.

### Kinetic cell proliferation assay

A375 cells were seeded in fresh medium in a flat‐bottomed 96‐well culture plate (Nunc, Thermo Fisher Scientific) at a density of 5,000 cells in 200 µL medium per well for 24 h before treatment. Then wild‐type cystatin C or its variants, (R24A,R25A)‐ and W106F‐cystatin C, were added at a concentration of 5 µm. The wells were covered with Hololids for 96‐well plates (Phase Holographic Imaging) and placed on the motorized HoloMonitor M4 stage immediately after addition of the different treatments. Time‐lapse imaging was conducted by the HoloMonitor M4, whereas holograms were generated from two random positions in each well every hour for 48 h according to the protocol for kinetic proliferation assay in the AppSuite software (Phase Holographic Imaging). Algorithms of the software automatically makes calculations for cell number and confluency from the images taken. Out of focus images were excluded manually by the user. Growth rates were calculated from the cell numbers obtained from AppSuite both for individual experiments and from mean cell numbers for all experiments, using the equation:

$$\text{growth}\;\text{rate}\;=\;\ln\left(N\left(t\right)/N\left(0\right)\right)/t,$$

where *N* is cell number at time *t*.

Cell doubling times were then calculated from mean cell numbers for all experiments, by the equation:

$$\text{doubling}\;\text{time}\;=\;\text{ln}\left(2\right)\text{/growth rate}$$

### Cell tracking

A375 cells (20 000) were seeded in a 6‐well cell culture plate (Sarstedt, Nümbrecht, Germany) and allowed to attach for 24 h. The medium was exchanged for fresh, without or with addition of 5 µm wild‐type cystatin C. The wells were covered with Hololids for 6‐well plates (Phase Holographic Imaging) and the plates were placed on the motorized stage of a HoloMonitor M4 in a standard 37°CCO_2_ incubator. Duplicate experiments were performed with 2 control wells with standard medium and 2 wells with addition of wild‐type cystatin C in each. Three positions in each well were chosen and images were captured every 5 min for 48 h. All images were visually inspected and out of focus images were excluded. The HStudio software (Phase Holographic Imaging) was used for cell tracking and analyses of the images. All cells in the first image were selected for analysis, except the cells that migrated out of the image area before their first division. The tracking continued until the cells divided, whereby the two resulting daughter cells were selected for further analysis and connected to the mother cell. This made it possible to calculate the cell cycle time for individual cells, as the new tracking started when the previous was completed and continued until next division was completed. With known timepoints for cell divisions and which cell the daughter cells originated from, a generation tree could be drawn using Excel (Microsoft).

### Analysis of cell morphology

For analysis of cell morphology, A375 cells were seeded in medium with and without cystatin C as described earlier for the kinetic cell proliferation assay. As a positive control for cell death, H_2_O_2_ was added at a final concentration of 100 µm, known to induce cell death in A375 cells [29]. Time‐lapse imaging was conducted and holograms were generated from two random positions in each well every hour for 24 h. Out of focus images were excluded manually by the user. The In‐depth Analysis function of the AppSuite software was used to analyze cell morphology. A scatter plot of cell area (µm), and average optical thickness (µm) was made at timepoints 0, 12, and 24 h using all cells from all wells and positions of each treatment. Phase holograms have earlier been used to study altered thickness and area, which are known properties of dead cells [28].

### Uptake of cystatin C variants in A375 cells

A375 cells were incubated for 6 h in standard medium or medium with addition of 1 µm of wild‐type cystatin C or (R24A,R25A)‐cystatin C in 6‐well plates. After incubation, cells were washed twice with PBS and incubated for 30 min with 250 µL lysis buffer (0.2% (v/v) Triton X‐100 (Sigma‐Aldrich) in PBS, supplemented with 5 mm benzamidinium hydrochloride, 15 mm NaN_3_, and 10 mm EDTA). Cystatin C was analyzed by ELISA as described elsewhere [17, 48]. The concentration of the cystatins was related to total protein concentration in the homogenates measured by Coomassie Protein Assay (Thermo Fisher Scientific).

### Measurement of cysteine cathepsin activity

A375 cells were seeded at a density of 500 000 cells·mL^−1^ in 12‐well plates and incubated for 24 h. Then 1 µm wild‐type or W106F‐cystatin C was added to the wells for 6 h. Control cells were cultured in standard medium. Cells were lysed with 250 µL lysis buffer. Portions of the lysates (5–10 μL) were added to black flat‐bottomed 96‐well microplates (Thermo Fisher Scientific) and mixed with 25 μL activation buffer (0.4 m sodium phosphate buffer, pH 6.5, with 4 mm EDTA, 4 mm DTT) and then adjusted to 100 µL with 0.01% Brij‐35 and the substrate Z‐Phe‐Arg‐NHMec (Bachem Feinchemikalien, Bubendorf, Switzerland) to a final concentration of 14 μm. The fluorescence was measured (Fluoroskan Ascent plate reader, LabSystems, Stockholm, Sweden) at excitation/emission wavelengths of 355/460 nm. All measurements were performed in triplicate in three individual experiments. The total protein content in the lysates was measured by Coomassie Protein Assay. The rate of substrate cleavage (arbitrary fluorescence units (FU)·min^−1^) was divided by the protein content (mg) to give the Z‐Phe‐Arg‐NHMec‐ase activity expressed in (FU·min^−1^·mg^−1^ of protein).

### Statistics

Statistics were calculated by Mann–Whitney rank‐sum test using the rstudio (RStudio, Boston, MA, USA) software.

## Conflict of interest

The authors declare no conflict of interest.

## Author contributions

HW planned and performed experiments, interpreted data, and drafted and wrote the manuscript; SH planned and performed experiments, interpreted data, and wrote the manuscript; MA planned and supervised experimental work, analyzed data, and co‐ordinated writing of the paper. The research described was supported by noncommercial, public academic funds. The funding sources had no involvement at any level in the design of the study and analysis or interpretation of the research results reported in the present paper.

## Data Availability

The data that support the findings of this study are available from the corresponding author [magnus.abrahamson@med.lu.se] upon reasonable request.
